# Severity of Hirsutism and Its Correlation With Hyperandrogenism: A Cross-Sectional Study in Erbil City

**DOI:** 10.7759/cureus.79406

**Published:** 2025-02-21

**Authors:** Dindar S Qurtas, Jwan H Ezzat

**Affiliations:** 1 College of Medicine, Hawler Medical University, Erbil, IRQ; 2 Department of Dermatology, Kurdistan Higher Council of Medical Specialties, Erbil Dermatology Teaching Center, Erbil, IRQ

**Keywords:** 17-hydroxyprogesterone, hirsutism, hyperandrogenism, progesterone, testosterone

## Abstract

Background: Hirsutism is defined as excessive terminal hair growth in females in androgen-dependent areas. Hyperandrogenism is a hormonal disease that clinically often presents as hirsutismus. Hirsutism is associated with high levels of androgens according to a few studies. This study is conducted to analyze this correlation in female individuals in Erbil City, Kurdistan Region, Iraq.

Methods: This case-control study included 100 hirsute females and their 100 non-hirsute counterparts. A thorough history was obtained for symptoms and conditions associated with hyperandrogenism. The severity of hirsutism was assessed with the modified Ferriman-Gallwey (mFG) score. Laboratory tests were performed to include thyroid function and androgen hormone levels.

Results: Most hirsute cases (98%) had mild and moderate hirsutism, and only 2% had severe hirsutism. Thyroid function tests were comparable in cases and controls, and only 4% of the hirsute study population had hypothyroidism. There were no significant differences in serum testosterone (total and free) between groups. But mean serum 17-hydroxyprogesterone values were higher in hirsute cases.

Conclusion: Elevated testosterone levels were present in only 2% of hirsute females and there were no statistically significant differences between the cases and control group. However, 17-hydroxyprogesterone was higher in hirsute females suggesting its possible involvement in the pathophysiology of hirsutism. Further studies that explore this relationship are recommended.

## Introduction

Hirsutism is defined as excessive terminal hair growth in women within androgen-dependent areas. It is a reflection of either an increase in circulating androgens or a heightened end-organ response to them. Increased production of androgens could be from the ovaries or adrenal glands, or occasionally from androgen-secreting tumors [1]. 

Hirsutism is diagnosed clinically by using a special visual scale called the modified Ferriman-Gallwey (mFG) score, where a total of ≥8 points is indicative of hirsutism. The mFG score assesses the density of terminal hair at nine distinct body regions, including the upper lip, chin, upper back, lower back, upper abdomen, arms, and thighs [2].

Hyperandrogenism may manifest clinically or biochemically. Clinical hyperandrogenism is defined as the presence of symptoms such as hirsutism, acne, and androgenic alopecia. Biochemical hyperandrogenism is defined as an elevated serum level of androgens, including total testosterone (TT), free testosterone (FT), androstenedione, dehydroepiandrosterone (DHEA), and the DHEA metabolite dehydroepiandrosterone sulfate (DHEAS) [3].

Although a proportion of hirsute women have elevated levels of circulating androgen, others do not. It has been established that there is a weak correlation between the degree of androgen excess and the manifested severity of hirsutism. Furthermore, it is still unclear which androgenic factor has the strongest influence on the mFG score [4]. Peripheral androgen metabolism also appears to influence the exhibition of hyperandrogenism [5].

Although hyperandrogenism has been a subject of much research, little has been studied about it regarding the Middle Eastern population, especially the population of Erbil, Kurdistan. Hirsutism is a multifactorial condition with genetic, environmental, and lifestyle factors influencing its presentation and severity, yet epidemiological information on the prevalence of hirsutism in this region is limited. There is a lack of some of this information that is necessary for achieving more accurate diagnosis and treatment specifically in this population [6]

The aim of this study is to evaluate the correlation of hirsutism severity with androgen hormone levels and clinical features of hyperandrogenism in hirsute women compared to healthy controls in Erbil, Kurdistan.

## Materials and methods

Study design and setting

This case-control study was performed in the Erbil Dermatology Teaching Center, Kurdistan Region, Iraq. The study was designed to evaluate the association between severity of hirsutism and biochemical markers of hyperandrogenism in women with and without hirsutism.

Study population and sampling method

A case-control study was performed on 100 consecutive hirsute women (cases) who were seen at the outpatient dermatology clinic with healthy female controls who did not have hirsutism. Controls were age-matched (±2 years) and BMI-matched (±2 kg/m^2^) to minimize potential confounding effects.

Inclusion and exclusion criteria

Cases were defined as females with mFG score ≥8. Pregnant or lactating women, women with premenarchal, postmenopausal, hormonal therapy, or hysterectomy history or diagnosed as having primary ovarian failure, as well as women who applied laser hair removal recently within the previous six months were excluded from the study. Controls were selected in the same clinic, mFG score <8, and with no androgen-related disorders history.

Data collection and assessments

Hirsutism was assessed using the mFG score in nine androgen-dependent sites (upper lip, chin, chest, upper back, lower back, upper abdomen, lower abdomen, thighs, and upper arms). In each of these areas, a score of 0 (absence of terminal hairs) through 4 (extensive terminal hair growth) was assigned [7]. Hirsutism severity was measured according to Abraham's classification as mild 8-16, moderate 17-24, and severe 24-36 [8].

Demographic and clinical data, including age, marital status, occupation, and family history of hirsutism or polycystic ovarian syndrome, were recorded after obtaining written informed consent. Gynecological and obstetric history (age menarche commenced, menstrual regularity, ovulation induction history, and in vitro fertilization (IVF) history) was documented. Dermatological manifestations, including acne, alopecia, and acanthosis nigricans, were noted. The medication history was documented, including glucocorticoids, supplements, and gym enhancers. We calculated body mass index (BMI) using the equation weight (kg)/height (m^2^).

Hormonal and biochemical assessment

Venous blood samples were obtained during the early follicular phase of the menstrual cycle. We measured serum TT, FT, and 17-OH progesterone using enzyme-linked immunosorbent assay (ELISA) kits. Thyroid-stimulating hormone (TSH), free triiodothyronine (T3), and free thyroxine (T4) levels were measured by a fully automated chemiluminescence immunoassay (Cobas e411 analyzer, Roche Diagnostics, Rotkreuz, Switzerland). All assays were validated and quality controlled internally, and calibrated using international standards.

Statistical analysis

Data were analyzed with SPSS 25.0 (IBM Corp., Armonk, NY). Continuous variables were presented as mean ± standard deviation (SD) and categorical variables were presented as frequency and percentage. Student’s t-tests were performed for the comparison of continuous variables between groups, and chi-squared or Fisher’s exact tests were used for categorical data. For three or more subgroups, a one-way analysis of variance (ANOVA) was used for comparisons. Based on data normality, Pearson’s or Spearman’s correlation coefficients were calculated. Multiple imputation techniques were used to handle missing data where appropriate. A p-value <0.05 was considered statistically significant.

Ethical considerations

Ethics approval was granted from the Kurdistan Higher Council for Medical Specialties (KHCMS) (Letter No. 14/241-2023). Informed written consent was achieved from all responders before explaining the objectives and procedures of the study. Data were treated confidentially, and participants were informed that they could withdraw at any time.

Data visualization and supplementary information

**Figure 1:**
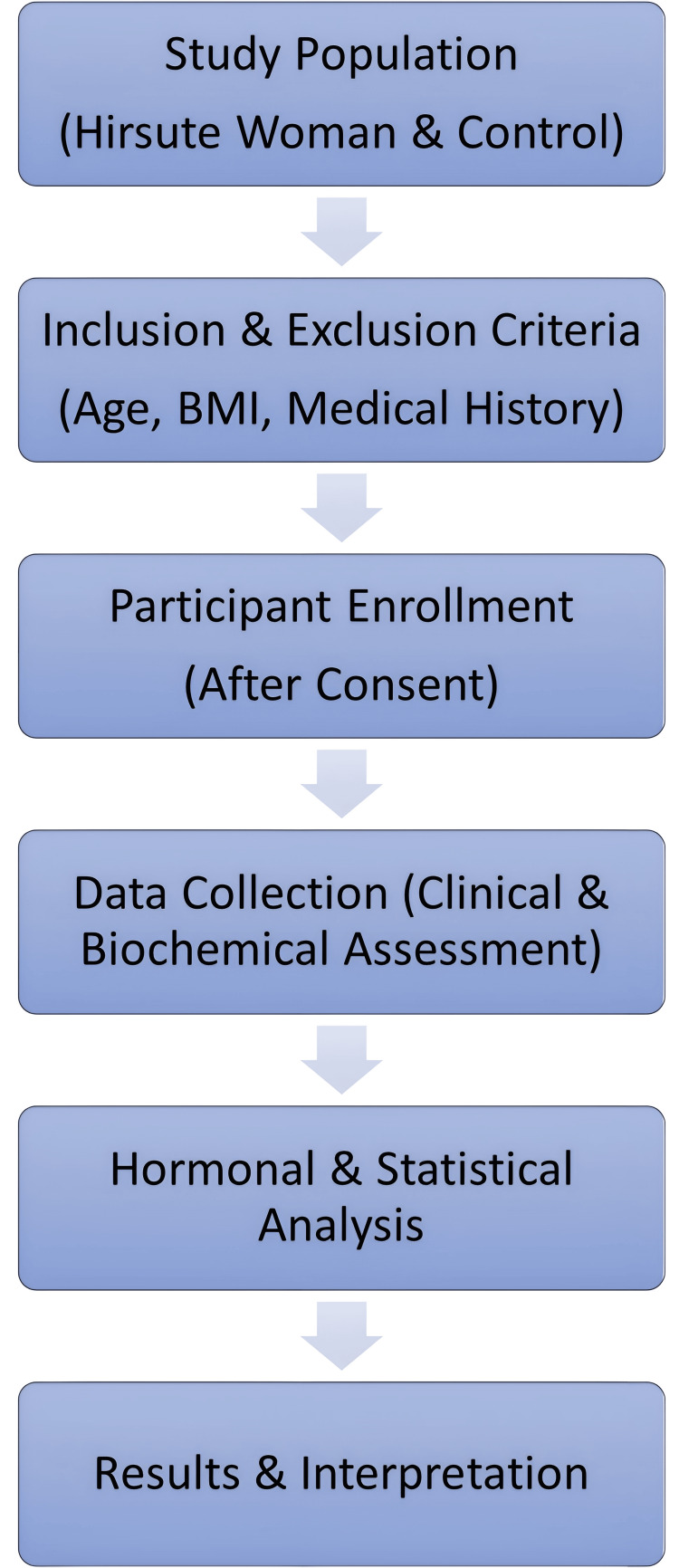
Study flowchart: selection criteria, data collection, and analysis framework Screening: Determining if participants fit in or out of involvement by selection criteria. Materials and methods enrollment: Informed consent was obtained to recruit eligible participants (hirsute women and healthy controls). Data collection: Clinical assessment using modified Ferriman-Gallwey (mFG) score, biochemical analysis of androgen levels, and demographic and medical history were documented. Data analysis using SPSS 25.0 for statistical evaluation of the correlation between hirsutism severity, clinical signs, and biochemical markers. Outcome: Associations between hirsutism severity and hyperandrogenism parameters are interpreted.

Participants were screened for eligibility based on selection criteria. Informed consent was then obtained to recruit eligible participants, including hirsute women and healthy controls. Data collection involved a clinical assessment using the mFG score, biochemical analysis of androgen levels, and documentation of demographic and medical history. Data analysis was performed using SPSS 25.0 to statistically evaluate the correlation between hirsutism severity, clinical signs, and biochemical markers. The results highlight the associations between hirsutism severity and hyperandrogenism parameters (Figure 1).

## Results

In this research, the average age of hirsute cases was 28.8±7.54 years compared to 27.2±4.9 years in controls (p=0.25). Furthermore, the two groups had similar ages of menarche (p=0.43). Hirsute ladies were more likely to be overweight (p=0.001), experience more menstrual irregularity (p=0.013), acne (p=0.004), and hair fall (p=0.001), and give a significant family history of polycystic ovary syndrome (PCOS) and hirsutism (p=0.001). The average mFG score in hirsute cases was 14.82±3.43 compared to 5.8±1.06 in controls (p=0.001). Only a few cases reported using medication with no apparent significant effect on mFG score (p=0.46) (Table 1).

**Table 1:** Clinical characteristics of hirsute cases and controls *p-Value <0.05 is considered to be significant. HTN, hypertension.

Characteristic	Index	Hirsute cases (n=100)	Controls (n=100)	p-Value*
Age (years)		28.8±7.54	27.2±4.9	0.25
Marital Status	Single	55	100	<0.001
	Married	45	0	
Occupation	Student	17	41	0.003
	Housewife	61	54	
	Public Employee	11	0	
	Self-employed	11	5	
Age of Menarche (years)		12.44±0.87	12.32±0.76	0.43
Regularity of Period	Regular	75	92	0.013
	Irregular	25	8	
Premenstrual Syndrome		29	31	0.8
Menstrual Character	Normal	74	90	0.14
	Oligomenorrhea	24	10	
	Polymenorrhea	1	0	
	Menorrhagia	1	0	
Parity	Nulliparous	56	98	<0.001
	Multiparous	44	2	
History of Induction of Ovulation		10	2	0.076
Presence of Acne		83	40	0.004
Presence of Hair Fall		55	10	0.001
Presence of Acanthosis Nigricans		13	2	0.059
Family History of Hirsutism		62	10	0.001
Family History of PCOS		30	4	0.001
Drug History	Anti-HTN	1	0	0.46
	Tonics	3	0	
	Herbals	3	0	
Weight (kg)		70.76±9.65	63.24±7.39	0.001
Height (cm)		159.7±4.58	159.14±2.78	0.42
BMI (kg/m^2^)		27.73±3.92	24.94±2.56	0.001
BMI Categories	Normal	27	65	0.003
	Overweight	49	26	
	Obese	21	9	
	Morbid Obese	3	0	
Modified Ferriman-Gallwey Score		14.82±3.43	5.8±1.06	<0.001
Hirsutism Severity	Normal (0-7)	0	100	<0.001
	Mild (8-16)	72	0	
	Moderate (17-24)	26	0	
	Severe (≥25)	2	0	

The distribution of terminal growth over the nine predefined androgenic areas of the body according to the mFG score is shown in Table 2. Compared to controls, the only site where terminal hair growth density did not differ in hirsute and non-hirsute was the upper lip region (p=0.12) (Table 2).

**Table 2:** mFG scores on androgen-sensitive areas of the body There were significant differences in every area but the upper lip. mFG, modified Ferriman-Gallwey. *p-Value <0.05 is considered to be significant.

Site	Cases (Mean±SD) (n=100)	Controls (Mean±SD) (n=100)	p-Value*
Upper Lip	2.66±0.68	2.41±0.62	0.12
Chin	2.32±0.76	0.36±0.59	<0.001
Chest	1.52±0.59	0.02±0.14	<0.001
Upper Abdomen	1.42±0.61	0.26±0.44	<0.001
Lower Abdomen	1.81±0.89	1.12±0.36	<0.001
Upper Arm	1.35±0.61	0.13±0.31	<0.001
Thighs	1.48±0.58	0.92±0.35	<0.001
Upper Back	1.01±0.11	0.02±0.14	<0.001
Lower Back	1.25±0.46	0.64±0.48	<0.001
Total (mFG Score)	14.82±3.43	5.8±1.06	<0.001

On hormonal evaluation of cases and controls, no significant differences were observed in mean serum levels of thyroid function status between them (p=0.4). On individual analysis of the subjects, only four hirsute cases (4%) had subclinical hypothyroidism. In general, there was a minimal discrepancy between serum TT and FT between the two groups. There was a mild elevation of mean serum levels of 17-OH progesterone more than normal, versus normal value among the control subjects (p=0.03) (Table 3).

**Table 3:** Hormonal assessment of hirsute cases and controls *p-Value <0.05 is considered to be significant.

Laboratory Test (Unit)	Reference Range	Cases (n=100)	Controls (n=100)	p-Value*
Serum TSH (µIU/mL)	0.27-4.2	2.52±1.4	2.63±0.97	0.64
Serum Free T3 (pmol/L)	3.1-6.8	5.37±0.82	5.06±0.93	0.32
Serum Free T4 (pmol/L)	12-22	14.41±2.0	15.16±2.08	0.25
Serum Total Testosterone (ng/mL)	0.06-0.82	0.38±0.25	0.32±0.26	0.13
Serum Free Testosterone (pg/mL)	0.4-7.1	4.18±2.26	2.41±1.21	0.07
Serum 17-OH Progesterone (ng/mL)	0.2-1.3	1.53±1.57	0.76±0.46	0.03

According to the mFG score of the hirsute cases, the severity was mild in 72 (72%) cases, moderate in 26 (26%) cases, and severe in two (2%) cases. The degree of severity of hirsutism was significantly different in terms of regularity of periods (p=0.001), menstruation character (p=0.001), premenstrual syndrome (p=0.02), and body weight (p=0.04). However, the association was statistically significant in more than half of them, resented by 60 (60%) cases of hirsute cases having regular menstrual periods; on the other hand, only two cases (2%) had irregular periods in severe hirsutism. Regarding the amount of menstrual bleeding, the majority, 74% (74 cases), had the normal amount of bleeding, and oligomenorrhea was observed in 24% (24 cases) of hirsute cases. Normal BMI was observed in only 27 hirsute cases (27%), and the rest had increased BMI (Table 4).

**Table 4:** Correlation of severity of hirsutism with menstruation and body mass *p-Value <0.05 is considered to be significant.

Variable		Hirsutism Severity (n=100)			p-Value*
		Mild (n=72)	Moderate (n=26)	Severe (n=2)	
Period Regularity	Regular	60	15	0	0.001
	Irregular	12	11	2	
Menstrual Character	Normal	61	13	0	0.001
	Oligomenorrhea	11	11	2	
	Polymenorrhea	0	1	0	
	Menorrhagia	0	1	0	
Perimenstrual Syndrome (kg/m^2^)		17	10	2	0.02
BMI	Normal	18	8	1	0.04
	Overweight	41	8	0	
	Obese	13	7	1	
	Morbid Obese	0	3	0	

**Figure 2:**
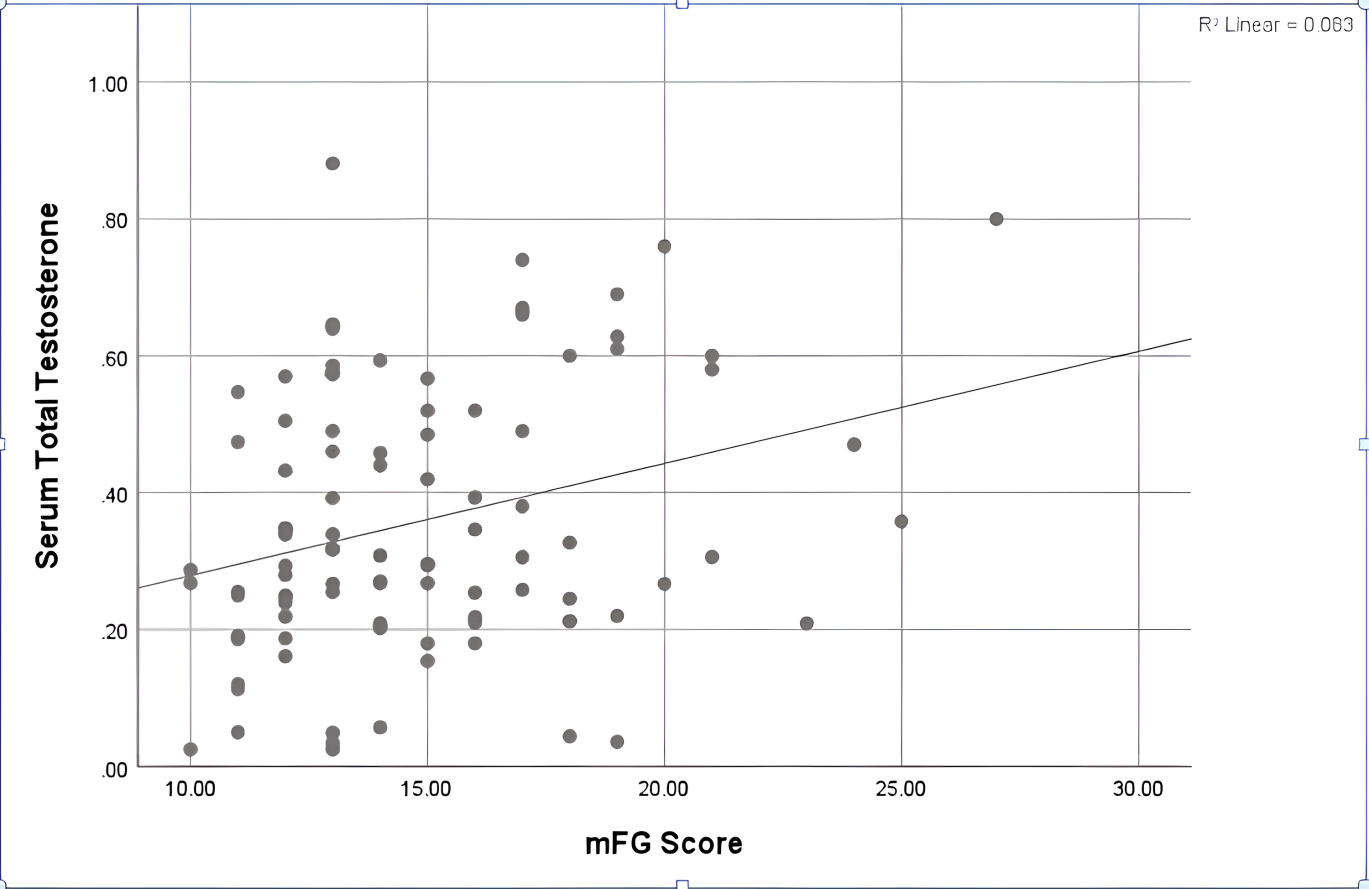
Correlation between mFG score and total serum testosterone in hirsute cases Serum total testosterone is measured in ng/mL. mFG, modified Ferriman-Gallwey.

Although the measured values of serum TT were within the normal range in the majority of cases (98%), only two cases (2%) had significant elevations in the serum TT, out of which only one case (1%) suffered from severe hirsutism. Only one case of hirsute females had high serum FT (Table 5). Despite these data, there was a trend toward higher mFG scores within the normal range of TT (p=0.006) (Figure 2).

**Table 5:** Mean serum testosterone levels according to hirsutism severity *p-Value <0.05 is considered to be significant. mFG, modified Ferriman-Gallwey.

Hirsutism Severity	mFG Score	Total Testosterone (ng/mL)				Free Testosterone (pg/mL)				p-Value*
		Normal (0.06-0.82)		High (≥0.82)		Normal (0.4-7.1)		High (≥7.1)		
		Frequency	Mean Value	Frequency	Mean Value	Frequency	Mean Value	Frequency	Mean Value	
Controls	≤7	99	0.32	1	0.8	98	2.78	2	7.5	0.006
Mild	8-16	71	0.33	1	0.85	71	1.98	1	7.8	
Moderate	17-24	26	0.43	0	NA	26	2.2	0	NA	
Severe	≥25	1	0.36	1	0.8	2	4.1	0	NA	

**Figure 3:**
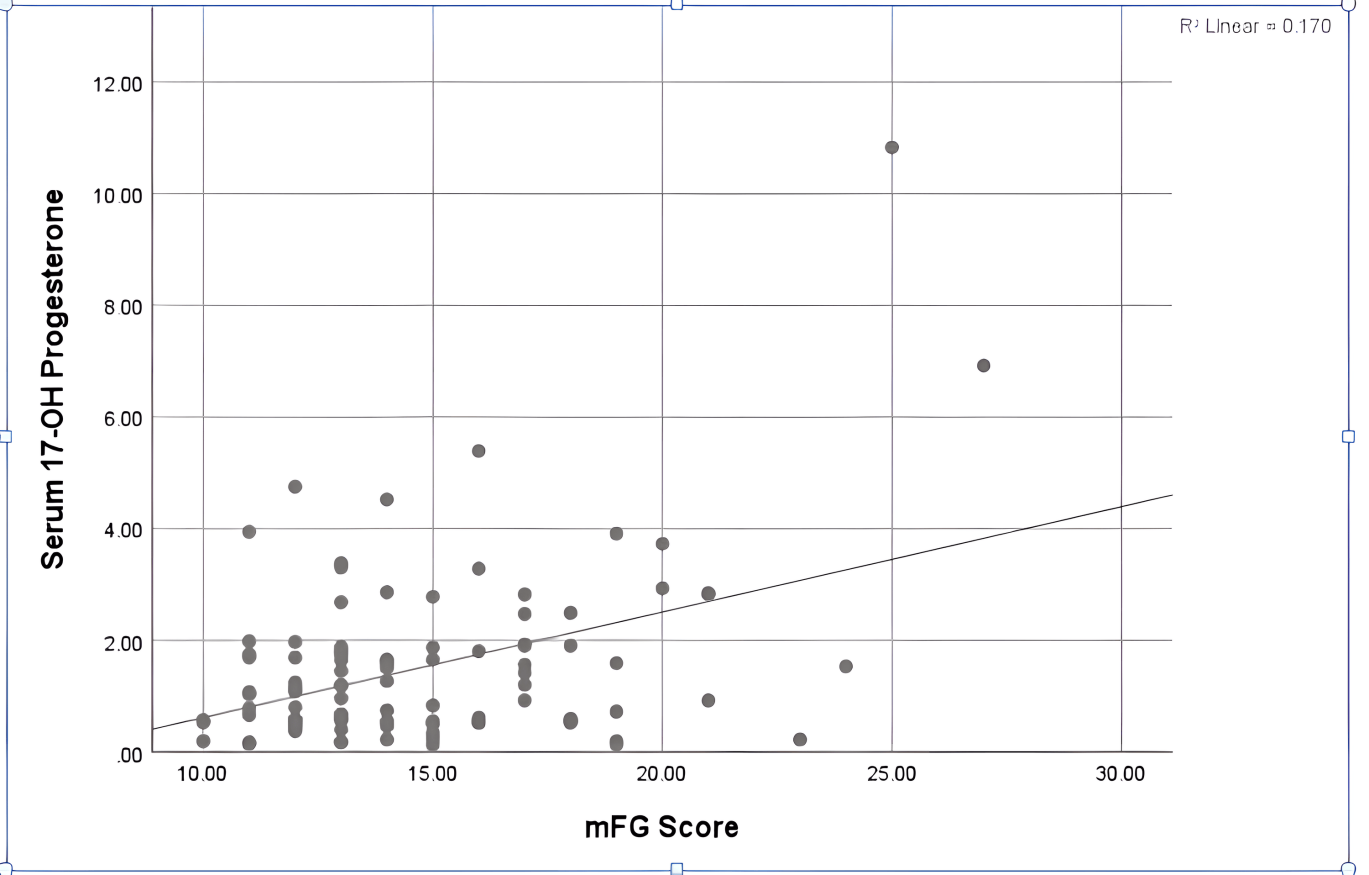
Correlation between mFG score and serum 17-OH progesterone in hirsute cases Serum 17-OH progesterone is measured in ng/mL. mFG, modified Ferriman-Gallwey.

A significant correlation was found between hirsutism severity and serum 17-OH progesterone level (p=0.03). About 19 (19%) hirsute cases had mildly elevated serum 17-OH progesterone levels, while only one case (1%) was found to have severe hirsutism together with high 17-OH progesterone (Table 6). The scatter plot shows the correlation between serum 17-OH progesterone and mFG score. There was a strong correlation (p=0.03) (Figure 3).

**Table 6:** Comparison of serum 17-OH progesterone levels in cases and controls *p-Value <0.05 is considered to be significant. mFG, modified Ferriman-Gallwey.

Hirsutism Severity	mFG Score	Serum 17-OH Progesterone (ng/mL)						p-Value*
		Normal (0.2-1.3)		Mildly Elevated (1.4-10)		High (>10)		
		Frequency	Mean Value	Frequency	Mean Value	Frequency	Mean Value	
Controls	≤7	96	0.7	4	2.2	0	NA	0.03
Mild	8-16	62	0.8	10	3.9	0	NA	
Moderate	17-24	18	1.2	8	3.2	0	NA	
Severe	≥25	0	NA	1	7.1	1	11.2	

## Discussion

Hirsutism is a genetically influenced condition and is characterized by excessive terminal hair growth in androgen-dependent areas, caused by a combination of genetic, hormonal, and environmental factors [1]. Etiologies include either an increase in circulating androgens or a heightened end-organ response to them. Increased production of androgens could be from the ovaries or adrenal glands, or occasionally from androgen-secreting tumors [2]. Hyperandrogenism manifests clinically as hirsutism, androgenic alopecia, and acne [8]. The prevalence of hirsutism is around 30% in premenopausal Iraqi women [9]. Evaluation of hirsutism can be made using the standard mFG score [6]. A common cutoff value of ≥8 is used if no population-specific studies recommend a different limit [6,7]. Significant variations in age, skin type, and race/ethnicity also play a role in redefining hirsutism in a given population [10,11]. Patients with endocrine organ-based dysfunction-related hirsutism are more likely to manifest an mFG score ≥15, while a normal or slightly elevated circulating levels of androgens may be detected in constitutional (dermatologic) hirsutism [12]. Regarding androgens, the circulating free, rather than TT, seems to exert a significant role in the transformation of vellus to terminal hair during puberty in the androgen-dependent areas of the female body [13]. BMI ≥30 is often seen in association with hirsutism because of increased conversion of androgen precursors to testosterone [14,15]. Measurement of 17-OH progesterone in hirsute women may be valuable in uncovering cases of late-onset congenital adrenal hyperplasia (CAH) [16]. Although uncommon, the incidence of CAH is around 1.9-3% in Mediterranean populations [17]. In this study, we investigated the correlation between the clinical signs and biochemical values of hyperandrogenism in hirsute women and non-hirsute controls in Erbil City, Kurdistan Region, Iraq. A statistically significant correlation was demonstrated between the severity of hirsutism according to mFG score and serum FT rather than TT, serum 17-OH progesterone, and other clinical features of hyperandrogenism. Despite the former mentioned association, while almost all (98%) hirsute cases had no elevation of their hormone levels in androgens, as mentioned in the other studies, values of mFG were more than 7, i.e. hirsutism have 7 times more elevated levels of serum FT [18]. Family history of hirsutism and PCOS among hirsute was observed to be significantly more than the non-hirsute. The majority of hirsute ladies in this study also exhibited other signs of hyperandrogenism like acne in 83% of cases and hair fall in 55% of cases which were much higher than non-hirsute subjects. These data regarding acne-associated hirsutism are slightly higher than those of a report published before [19]. In this study, no correlation between thyroid dysfunction and hirsutism was seen and the same observation was found among non-hirsute cases; however, in some other studies, some percentage (16-17%) of hirsute cases of PCOS had thyroid dysfunction [20,21]. Recent studies demonstrated various findings. In a study conducted on hirsute women in Basrah city, FT, rather than TT or DHEAS, was found to correlate with the severity of hirsutism [22]. In Filipino women, FT was likely to be elevated if the mFG score was >7. In 80 Iraqi women with PCOS, higher levels of testosterone were recorded compared to healthy controls, with the magnitude of elevation of serum testosterone being proportionate to the severity of manifested hirsutism [23]. Conversely, a study in Kalar did not show these findings, and no correlation to any hormonal disturbance could be demonstrated in the study group [24]. In a study evaluating serum FT in hirsute PCOS women in Soran City, the highest levels were recorded in the youngest age group (18-20-year-olds) [25]. In Kirkuk City, a study of 1000 young lean female students with no underlying ovarian dysfunction or menstrual irregularity, i.e., non-PCOS etiology of hirsutism, has shown a significant correlation between hirsutism severity and serum FT, DHEAS, androstenedione, and sex hormone-binding globulin (SHBG). A strong family history was also uncovered in a significant proportion of cases [26]. It is recommended that the approach to the management of hirsutism should be based on a thorough and detailed history and physical examination of the affected women [27,28]. The higher quartiles of mFG scores are generally expected to be related to significant pathology [29,30]. Thus, according to the results of our study, especially in resource-limited health systems, over-investigating the cause of apparently mild-moderate hirsutism with costly hormonal assays may not be necessary, and can be avoided with careful selection of diagnostic testing according to the clinical probability determined by the dermatologist and/or endocrinologist.

An important shortcoming of our study is the low number of severe hirsutism cases, which limits statistical power and generalizability. Further investigation will require a large sample with more severe cases. Moreover, the cross-sectional design of the study does not allow conclusions on a cause-effect basis; longitudinal studies measuring hormonal changes over time could clarify the natural evolution of hirsutism.

Another significant limitation was the lack of DHEAS measurement which would have given additional information about the adrenal contribution to hyperandrogenism. To enhance diagnostic accuracy, future studies should incorporate a broader hormonal panel, potentially including androstenedione and SHBG [26,30]. Moreover, the investigation of potential metabolic confounders, including insulin resistance and obesity-related hyperinsulinemia, may give a better understanding of the pathophysiology of hirsutism.

Recommendations for clinicians in practice

In approaching hirsutism, clinicians should utilize a stepwise diagnostic method, starting with detailed history-taking and physical examination, reserving extensive biochemical testing for patients with specific features on history and physical examination [27,28]. This approach should be tailored to the patient due to the frequency of mild to moderate hirsutism with normal androgen levels based on family history, BMI, and metabolic risk. Where there is severe or progressive hirsutism, specific endocrine evaluation including 17-OH progesterone and DHEAS should be the main focus.

## Conclusions

This adds to the body of literature documenting regional patterns of hirsutism/hyperandrogenism, particularly from the Middle Eastern population. Our results underscore FT and 17-OH progesterone being relevant biochemical markers for hirsutism evaluation results in a more clinically relevant, economical, and lower-cost approach. Although the small number of severe cases limits the generalizability of findings, the study highlights the need for context-specific diagnostic strategies and further research to improve clinical guidance. Future studies should seek to validate these findings across larger cohorts, integrate more biochemical markers, and continue to elucidate the genetic and metabolic basis of hirsutism in various populations. By taking into account these aspects, the performance in diagnosing and treating hirsutism could be bettered, and health outcomes improved.

## References

[1] Cussen L, McDonnell T, Bennett G, Thompson CJ, Sherlock M, O'Reilly MW (2022). Approach to androgen excess in women: Clinical and biochemical insights. Clin Endocrinol (Oxf).

[2] Khare VR, Sinha B, Sengupta N (2024). Practise updates: Diagnosis and management of idiopathic hirsutism. Indian J Endocrinol Metab.

[3] Heijboer AC, Hannema SE (2023). Androgen excess and deficiency: Analytical and diagnostic approaches. Clin Chem.

[4] Darjani A, Alizadeh N, Gharaei Nejad K, Eftekhari H, Rafiei R, Kazemi H, Rafiei E (2023). Testosterone or dihydrotestosterone: What should be evaluated in hirsutism?. Ir J Med Sci.

[5] Armata I, Prakash A (2024). An update on the assessment and management of hirsutism. Obstet Gynaecol Reprod Med.

[6] Badr F, Chattha AJ (2024). Is there a difference in hirsutism score in adolescents with polycystic ovary syndrome on the basis of ethnicity and race?. J Pediatr Adolesc Gynecol.

[7] Mauvais-Jarvis P, Kuttenn F, Mowszowicz I (1981). Clinical and biological assessment of hirsutism. Hirsutism.

[8] Sharma A, Welt CK (2021). Practical approach to hyperandrogenism in women. Med Clin North Am.

[9] Sharquie KE, Al-Khafaji KA (1992). The prevalence of hirsutism in Iraqi females. Ann Saudi Med.

[10] Javorsky E, Perkins AC, Hillebrand G, Miyamoto K, Boer Kimball A (2014). Race, rather than skin pigmentation, predicts facial hair growth in women. J Clin Aesthet Dermatol.

[11] Guo Z, Jin F, Chen S, Hu P, Hao Y, Yu Q (2023). Correlation between biochemical and clinical hyperandrogenism parameter in polycystic ovary syndrome in relation to age. BMC Endocr Disord.

[12] Mody A, Shinkai K (2021). Addressing important knowledge gaps about the disease burden of hirsutism. Int J Womens Dermatol.

[13] Al Kindi MK, Al Essry FS, Al Essry FS, Mula-Abed WA (2012). Validity of serum testosterone, free androgen index, and calculated free testosterone in women with suspected hyperandrogenism. Oman Med J.

[14] Aswini R, Jayapalan S (2017). Modified Ferriman-Gallwey score in hirsutism and its association with metabolic syndrome. Int J Trichology.

[15] Lumezi BG, Berisha VL (2021). Investigation of body mass index, insulin resistance and diabetes in patients with hirsutism. Postepy Dermatol Alergol.

[16] Witchel SF (2017). Congenital adrenal hyperplasia. J Pediatr Adolesc Gynecol.

[17] Demirci T, Cengiz H, Varım C, Çetin S (2020). The role and importance of auxiliary tests in differential diagnosis in patients with mildly high basal 17-OH-progesterone levels in the evaluation of hirsutism. Turk J Med Sci.

[18] Ilagan MK, Paz-Pacheco E, Totesora DZ, Clemente-Chua LR, Jalique JR (2019). The modified Ferriman-Gallwey score and hirsutism among Filipino women. Endocrinol Metab (Seoul).

[19] Matheson E, Bain J (2019). Hirsutism in women. Am Fam Physician.

[20] Kamrul-Hasan AB, Aalpona FT, Mustari M (2020). Prevalence of thyroid dysfunction and thyroid autoimmunity in polycystic ovary syndrome: A multicenter study from Bangladesh. Thyroid Res Prac.

[21] Zwain ZM, Aziz MK (2016). Polycystic ovarian syndrome and thyroid disorders. In J Tech Res App.

[22] Hussein RN, Al Hamdi KI, Mansour AA (2021). Association between biochemical hyperandrogenism parameters and modified Ferriman-Gallwey score in patients with hirsutism in Basrah (Southern Iraq). Postepy Dermatol Alergol.

[23] Alsaadi YL, Mohamad BJ (2019). Prevalence of hyperandrogenism in Iraqi women with polycystic ovary syndrome. Iraqi J Sci.

[24] Jarallah B, Adil H, Palani A (2023). Comparison of modified Ferriman-Gallwey score and biochemical parameters in the estimation of the prevalence of idiopathic hirsutism among women in the Kurdistan region. Baghdad J Biochem Appl Biol Sci.

[25] Jalil PJ, Shnawa BH, Ahmed MH (2023). Association of free testosterone, glucose level and obesity among women with polycystic ovary syndrome in Soran city, Kurdistan-Iraq. Clin Invest Ginecol Obstet.

[26] Ibrahim RO, Ali NK, Ali IS (2023). Clinical and biochemical evaluation of hirsutism in young, lean girls from Kirkuk City, Iraq: A cross-sectional study. Al-Rafidain J Med Sci.

[27] Unluhizarci K, Hacioglu A, Taheri S, Karaca Z, Kelestimur F (2023). Idiopathic hirsutism: Is it really idiopathic or is it misnomer?. World J Clin Cases.

[28] Unluhizarci K, Gokce C, Atmaca H, Bayram F, Kelestimur F (2004). A detailed investigation of hirsutism in a Turkish population: Idiopathic hyperandrogenemia as a perplexing issue. Exp Clin Endocrinol Diabetes.

[29] Hirschberg AL (2023). Approach to investigation of hyperandrogenism in a postmenopausal woman. J Clin Endocrinol Metab.

[30] Mahajan VK, Singh Chauhan P, Chandel M (2021). Clinico-investigative attributes of 122 patients with hirsutism: A 5-year retrospective study from India. Int J Womens Dermatol.

